# Excess Food Availability Can Have Detrimental Effects on Planktotrophic Larvae: Evaluation of the Quantitative Feeding on Sea Urchin *Paracentrotus lividus* Larval Rearing

**DOI:** 10.1155/anu/6467396

**Published:** 2026-07-27

**Authors:** Luca Grosso, João Sousa, Pedro M. Santos, Francisco Azevedo e Silva, Sílvia Lourenço, Pedro M. Félix, Ana Pombo, Arnold Rakaj

**Affiliations:** ^1^ Laboratory of Experimental Ecology and Aquaculture, Department of Biology, University of Rome Tor Vergata, Via Cracovia 1, Rome 00133, Italy, uniroma2.it; ^2^ MARE—Marine and Environmental Sciences Centre/ARNET—Aquatic Research Network, School of Tourism and Maritime Technology (ESTM), Polytechnic Institute of Leiria, Peniche 2520–630, Portugal, ipleiria.pt; ^3^ MARE—Marine and Environmental Sciences Centre/ARNET—Aquatic Research Network, Faculty of Sciences, University of Lisbon, Lisbon 1749–016, Portugal, ulisboa.pt

## Abstract

Larval rearing in echinoculture still has room for optimization in order to reach economical soundness and commercial‐scale production. One of the key factors in larval feeding is to provide larvae the necessary energy for metamorphosis into juveniles. *Paracentrotus lividus* (Lamarck, 1816) is a meroplanktonic species with a planktotrophic larval stage, relying entirely on phytoplankton as a food source due to its limited maternal yolk reserves. While the importance of optimal microalgal diets in maximizing larval yields is well established, there is a lack of comprehensive studies on the ideal feed ratio for *P. lividus* larval rearing. The present study aimed to fill this knowledge gap in this Atlanto‐Mediterranean sea urchin, assessing the effects of different food ratios on its planktonic development, to shed light on the existence of optimal food quantity to adopt. We tested three different concentrations (low, medium, and high ratios) of a mixed microalgal diet (*Isochrysis galbana*, *Chaetoceros calcitrans*, and *Rhodomonas lens*) which were doubled on each transition to the subsequent larval development stage. The results showed that the food quantity significantly affected the survival rate, development, lifespan, and metamorphosis success of pluteus, indicating the range from 1500 to 12000 cells·mL^−1^ as optimal, with lower yields occurring both at higher and lower feeding ratios.

## 1. Introduction

Sea urchin gonads (sea urchin roe) are considered a delicacy due to their distinctive flavor and excellent nutritional value. As a result, sea urchin roe has become one of the most sought‐after and expensive marine products on the market [1, 2]. *Paracentrotus lividus* is the most exploited echinoid species in the Mediterranean Sea, where reported catches increased from 66 t in 2013 to 387 t in 2019 [3, 4]. This increasing exploitation pressure has contributed to the rapid decline of natural populations, with recent surveys in some heavily impacted Italian areas reporting extremely low mean densities of ~0.2 ind·m^−2^ and only 6% of individuals reaching commercial size [4]. These data highlight the urgent need to reduce harvesting pressure on wild stocks and to identify sustainable supply alternatives for this commercially valuable species. In this context, aquaculture could be an effective tool in both meeting the market demand and restocking overexploited populations. However, the long‐term rearing cycles to reach the market size (around 2–4 years for *P. lividus* [5]) are still the main issue to be solved before the echinoculture reaches the economical soundness and commercial scale production [6, 7].

To face these challenges, new production solutions and cutting‐edge technologies are emerging. Recently, Rakaj et al. [8] proposed an innovative production strategy, termed raking, enabling the production of sea urchin caviar as an alternative to whole gonads, which represent the traditional extractive product predominantly marketed for sushi consumption. This echinoculture approach does not require the sacrifice of the animals at the end of the production cycle, thereby offering a solution to both the traditional constraints of the long rearing period to reach the market size and the exploitation of wild stocks. However, despite the raking advantages over traditional methods, biological constraints may necessitate the periodic renewal of sea urchin batches after multiple spawning cycles. Therefore, advancing full‐life‐cycle aquaculture, where the entire production process (from fertilization to market‐sized individuals) occurs in captivity, is essential to making echinoculture fully independent of wild fisheries.

One of the main challenges to completing the full life cycle in echinoculture remains the high mortality rates during the initial rearing phases, with cumulative losses reaching up to 95% of the initial egg stock under standard rearing conditions [9–11]. On this line, several studies have investigated larval rearing protocols under controlled laboratory conditions, and increasing efforts are being made to scale these approaches to larger culture volumes and pilot‐scale production systems [12–19].

In particular, food is a key factor for ensuring that larvae receive the necessary energy to metamorphose into benthic juveniles. In fact, *P. lividus* has planktotrophic larvae (ranging between 200 and 1000 μm in length) with little maternal yolk reserves [20], so they fully rely on phytoplankton as a food source to complete their development [21]. Providing optimal microalgae diets is, therefore, fundamental to overcome bottlenecks in larval rearing [22]. Previous investigations highlighted that the feeding quality (the provision of specific algal strains) may strongly influence larval survival rate and postsettlement yields [9, 23–27].

The choice of the microalgae strains has a direct (nutritional properties) impact on growth, competence (capacity for settlement), and survival [28]. To achieve maximum larval growth and survival rates per unit of culture effort, it is desirable to supply a mix of multiple microalgae species with optimal nutritional value to meet *P. lividus* requirements [10, 29–32]. Flagellates are widely used due to their high content of essential polyunsaturated fatty acids, particularly DHA, which are crucial for larval growth and survival [9, 33]. Diatoms contribute structural lipids, sterols, and silica, supporting skeletal growth and normal morphogenesis [10, 26]. Cryptophytes are characterized by high protein content and a balanced fatty acid profile, which can enhance larval condition and developmental performance [28, 34]. The combination of these microalgae is generally considered effective in meeting the nutritional requirements of planktotrophic sea urchin larvae.

Although a large body of research has focused on qualitative feeding, allowing the identification of suitable algal strains, very little is known about the effect of food quantity for this sea urchin species. In planktotrophic echinoderm larvae, feeding is mediated by ciliary suspension‐feeding mechanisms, and particle capture, rejection, and ingestion rates are influenced by food‐particle concentration, larval morphology, and the functional capacity of the ciliated band [35]. However, the extent to which *P. lividus* larvae can actively regulate food intake under different microalgal concentrations remains poorly characterized. This is particularly relevant because recent studies have shown that food availability can act as a major developmental cue in echinoid larvae, inducing phenotypic plasticity in feeding structures and larval morphology. In *P. lividus*, *Arbacia lixula*, and *Strongylocentrotus purpuratus*, larvae exposed to different food levels showed plastic responses in skeletal arm development, with light conditions modulating the magnitude of this response [36]. More broadly, phenotypic plasticity in ciliated‐band length has been documented across feeding echinoderm larvae [37], and food‐induced plastic responses in echinoid larvae can be inducible and reversible throughout development [38]. Although the appropriate food quantity to administer at each larval developmental stage has been investigated in other sea urchin species [25, 26, 39], it remains unknown for *P. lividus*. Hence, assessing the suitable feeding amount for each larval stage is crucial to optimize food supply and improve larval survival and metamorphosis rates in aquaculture. Previous studies on *P. lividus* larval rearing have used a wide range of microalgal concentrations, typically spanning from a few 100 to several 1000 cells·mL^−1^, depending on the larval stage and rearing conditions. Feeding regimes commonly range between ~500 and 5000 cells·mL^−1^ during early pluteus stages, with progressive increases up to 10,000–15,000 cells·mL^−1^ at later developmental stages to support growth and competence [9, 10, 14, 29]. This broad variability underscores the need to define optimal feeding regimes.

Hence, the aim of this study was to shed light on the optimal food quantity for *P. lividus* larval development to optimize its hatchery production. With this purpose, we tested three different concentrations (low, medium, and high ratios) of a mixed microalgae diet on larval survivorship and development. *Isochrysis galbana*, *Chaetoceros calcitrans*, and *Rhodomonas lens* were selected among the most commonly used and effective microalgae in *P. lividus* larval rearing, which achieved optimal results in previous studies and differed greatly in cell size and nutritional profiles [10, 14, 18, 28, 29, 40]. Once larvae reached competence, the effects of the three experimental food ratios were assessed also in terms of metamorphosis yields in juveniles.

## 2. Materials and Methods

### 2.1. Broodstock Collection

Adult *P. lividus* specimens (40 individuals; mean weight: 58.78 ± 4.11 g; mean ± SD) were collected manually in the intertidal zone of Porto Batel (Peniche, Portugal; 39°19′11″N; 009°21′21″W) in May 2023. At the time of collection, the seawater temperature at the sampling site was ~16 ± 1°C.

Once in the laboratory of Cetemares (MARE—Polytechnic of Leira), the broodstock was acclimated and maintained in a 100 L recirculating aquaculture system, equipped with a protein skimmer (Tunze DOC 9460) and biological filtration (Moving Bed Biofilm Reactor MBBR, SCUBLA). The sea urchins fasted for a week to void their gut content, and the room temperature was set to 18 ± 1°C to reduce the risk of spontaneous spawning during this period. Physicochemical environmental parameters (temperature, salinity, pH, and dissolved oxygen) were daily monitored with a YSI Professional Plus handheld multiparameter sonde (YSI Incorporated, Yellow Springs, U.S.A.). During this period, the environmental parameters were maintained within the following ranges (mean ± SD): water temperature 18 ± 1°C, salinity 35, pH 7.98 ± 0.59, and dissolved oxygen 7.98 ± 0.45 mg·L^−1^. Ammonia (detention limits 0–3.0 ± 0.01 mg·L^−1^) and nitrite (detention limits 0–30 mg·L^−1^ ± 0.01 mg L^−1^) were monitored daily with a HANNA HI 83203 (HANNA Instruments, Woonsocket, U.S.A.) multiparameter bench photometer, and a water change (50% of the total volume) was performed every day to maintain the optimal parameter values in the maintenance system.

### 2.2. Fertilization and Larval Rearing

Before spawning induction, the sea urchins were washed with previously autoclaved seawater (121°C for 25 min) and subsequently injected with 1 mL of 0.5 M KCl into the celom through the peristomial membrane to induce gametes deposition. Oocytes were collected by placing females on a 100 mL beaker with the aboral region facing downward in contact with seawater. Egg quality was checked by stereoscope observations, and the eggs of 10 females were gathered in a single beaker by discarding females that spawned gametes not round, with immature forms or debris. Sperm was collected “dry” directly from the gonopores of 10 males using a Pasteur pipette and was kept on melting ice until use. The total egg quantity was estimated by collecting five subsamples of 1 mL egg culture and counting them with the Sedgewick Rafter counting chamber under a Zeiss Axio Lab.A1 microscope (Carl Zeiss, Oberkochen, Germany) with × 10 magnification. The sperm concentration and mobility were assessed using a Neubauer counting chamber under × 400 magnification. Once the concentration and vitality of gametes were checked, fertilization was performed by diluting 100 µl of sperm in 50 mL of seawater and adding 1 mL of this solution to 2000 mL of egg suspension (1000 eggs/mL) [41]. Twenty minutes after this procedure, the ratio of fertilization success was evaluated by observing the presence of the fertilization envelope, a membrane around the eggs. Then, the zygotes were reared in a 20 L tank (at a stocking density of 50 cells·mL^−1^), and degenerated embryos that daily gathered at the bottom were regularly removed. In this way, after 48 h, only larvae capable of free swimming in the water column were used for the further experimental steps, discarding those settled on the bottom of the tank. These larvae were equally divided into nine 50 L cylindroconical tanks (42cm × 70 cm, diameter × height) arranged in a Latin square design to test three experimental conditions. The cylindroconical tanks were filled with seawater previously filtered (2 μm) and UV‐treated. Continuous aeration was supplied via silicone tubing connected to fine‐bubble air stones placed at the base of each tank, ensuring homogeneous mixing and adequate oxygenation while minimizing mechanical stress on the larvae. Under these conditions, 4‐arm larvae were reared at an initial density of ~4.4 larvae·mL^−1^ (Table S1),~200,000 of larvae per tank, at a temperature of 20°C and 12 h:12 h photoperiod. Every day, the water parameters (temperature (°C), salinity, pH, dissolved oxygen (mg·L^−1^), ammonia, and nitrite concentrations (mg·L^−1^) were monitored, and 50% of the water volume was renewed with a 60 µm mesh screened siphon.

### 2.3. Feeding Experiment and Microalgae Cultures

*I. galbana*, *C. calcitrans*, and *R. lens* were selected among the most commonly used microalgae in *P. lividus* larval rearing. The microalgae cultures were grown in 1 or 2 L glass flasks with autoclaved seawater enriched with a commercial F/2 culture medium (Nutribloom Plus, Necton, Portugal). They were maintained at 20°C in a temperature‐controlled room with continuous phytostimulant light and gentle aeration. Silicate was supplemented exclusively in diatom cultures at 1 mL·L^−1^ using Nutribloom Silicates Solution (Necton, Olhão, Portugal; Si 0.3% w/w). Microalgae species were cultured using a semicontinuous method, with 20%–50% of the culture volume renewed two to three times per week, in order to maintain the cultures in the exponential growth phase for the entire duration of the experiment.

Prior to the feeding experiment, the relationship among optical density (OD), biomass, and cell density was determined in five different algal concentrations (1:1; 1:2; 1:4; 1:6, 1:8; microalgal culture:seawater), which were obtained by serial dilution for each microalgal culture in the exponential growth phase.

i. OD was calculated by evaluating the maximum absorbance between 550 and 800 nm using a UV–visible spectrophotometer (Jasco V‐630, USA). Then, the maximum absorbance wavelength was used to calculate the OD in each algal‐diluted culture [42].
ii. Cell number of each dilution was determined by cell counting through a Neubauer counting chamber under the microscope.
iii. Dry biomass was determined by filtering 10 mL of algal cultures onto a preweighed (W0) glass microfiber (GF/C) filter (pore size 0.45 μm) following dry‐weight estimation by the gravimetric method [43].

These measurements were used to construct linear regression models correlating OD, dry biomass, and cell density for each algae strain. The model equations allowed the daily quantification of dry biomass per milliliter of algal culture and the calculation of the relative contribution of each microalgal strain to the experimental diets (low, medium, and high):

C.calcitrans:y=0.40.03x+R2:0.99,

I. galbana:y=0.40.02x+ R2=0.99,

R.lens:y=0.50.04x+R2:0.98,

where *y* represents dry biomass (μg·mL^−1^) and *x* represents the OD value.

The experimental diet protocol was started when a fully developed gut was observed in larvae (48 h postfertilization). Larvae were fed daily with the same mixture of microalgae (*I. galbana*, *C. calcitrans*, *and R. lens*), supplied in equal dry biomass proportions (~33.3% each), under three different feeding regimes (low ratio, medium ratio, and high ratio; Table 1). The selected food ratios were established through preliminary feeding trials performed under comparable rearing conditions within the same experimental setup. Each feeding regime was tested in three replicas. During larval development, the administered food concentrations were doubled every ontogenetic transition from 4‐arm pluteus to the competence stage [26].

**Table 1:** Experimental food ratios.

Experimental food ratios	4‐arm pluteus	6‐arm pluteus	8‐arm pluteus	Competence
Low ratio (LR)	500 cells·mL^−1^ 0.04 µg·mL^−1^	1000 cells·mL^−1^ 0.08 µg·mL^−1^	2000 cells·mL^−1^ 0.16 µg·mL^−1^	4000 cells·mL^−1^ 0.32 µg·mL^−1^
Medium ratio (MR)	1500 cells·mL^−1^ 0.12 µg·mL^−1^	3000 cells·mL^−1^ 0.24 µg·mL^−1^	6000 cells·mL^−1^ 0.48 µg·mL^−1^	12,000 cells·mL^−1^ 0.96 µg·mL^−1^
High ratio (HR)	5000 cells·mL^−1^ 0.40 µg·mL^−1^	10,000 cells·mL^−1^ 0.80 µg·mL^−1^	20,000 cells·mL^−1^ 1.60 µg·mL^−1^	40,000 cells·mL^−1^ 3.2 µg·mL^−1^

*Note:* Low, medium, and high ratio are the three tested concentrations of a mixed microalgal diet (*Isochrysis galbana*, *Chaetoceros calcitrans*, and *Rhodomonas lens*). The algal concentrations are expressed as cells·mL^−1^ and the corresponding biomass in dry weight (µg·mL^−1^) are reported below the cell‐density values.

The larvae survival rate and development were assessed every 2 days by six subsamples of 10 mL collected in each replica. The rearing water was stirred to ensure a homogeneous distribution of larvae before sample collection, and then Lugol’s fixative was used to fix and preserve the samples for counting larvae in each developmental stage.

Larval survival was evaluated as the mean of larvae·mL^−1^ in the six subsamples of each replica, excluding the larvae extracted daily for each assessment. The larvae were divided into the developmental stages following the criteria described in Formery et al. [44] and illustrated in Figure S1: Stage I, four‐arm stage (appearance of the left and the right anterolateral arms); Stage II, six‐arm stage (appearance of a third pair of arms, called the left and the right posterodorsal arms, which formed in a posterior and dorsal position relative to the anterolateral arms); Stage III, eight‐arm stage (appearance of fourth and last pair of arms, called the left and the right preoral arms, which developed posterior to the anterolateral arms, on the ventral side of the oral hood); Stage IV, competence stage (appearance of rudiment, which is a structure on the left side of the stomach, corresponding to the anlage of the future sea urchin adult); Stage V, metamorphosed juveniles.

The culture development stage was changed when at least 50% of the larvae reached the subsequent stage.

Finally, metamorphosis assessments were performed when at least 75% of larvae reached competence and the rudiment developed inside the pluteus. Plastic substrates (bio‐balls) were conditioned by exposure to seawater enriched with *Phaeodactylum tricornutum* for 10 days to allow a diatom‐dominated biofilm on their surfaces. The conditioned substrates were then placed in the rearing tanks to induce the pluteus’s settlement and metamorphosis. Otherwise, in the conditions where larvae did not reach the competence, the bio‐balls were placed on the 30th day of rearing.

When competent larvae disappeared from the water columns, 5 L of seawater was removed and replaced in each tank with a solution of 5% w/v (weight/volume) concentration of KCl to reach a final concentration of 0.5% w/v, which was used as a detachment agent to facilitate grading and counting of metamorphosed juveniles, following the protocol of Hagen [45].

### 2.4. Statistical Analysis

The effects of food ratio on larval survival and metamorphosis were analyzed using analysis of variance (ANOVA) and independent samples *t*‐tests. Prior to analysis, data were assessed for normality (Shapiro–Wilk test) and homogeneity of variances (Levene’s test and *F*‐statistic). When ANOVA results indicated statistically significant differences (*p* < 0.05), Tukey’s post hoc test was applied for pairwise comparisons between groups [46]. Effect sizes were quantified using Cohen’s *d*, which revealed substantial differences between groups, with magnitudes interpreted as follows: small (|*d*| = 0.2), medium (|*d*| = 0.5), and large (|*d*| ≥ 0.8). To evaluate the combined effects of time and food ratio on larval development, survival, and metamorphosis, a two‐way PERMANOVA (permutational multivariate ANOVA) was conducted. This analysis tested both main effects and their interaction term, providing an insight into their combined influence on larval performances. All statistical analyses were performed using PAST 3.0 [47].

## 3. Results

The gamete collection and the adopted fertilization procedure resulted in highly effective fertilization, achieving a fertilization success rate of ~92%. After 48 h, well‐developed pluteus larvae were obtained and subsequently utilized for the following experimental steps.

**Figure 1:**
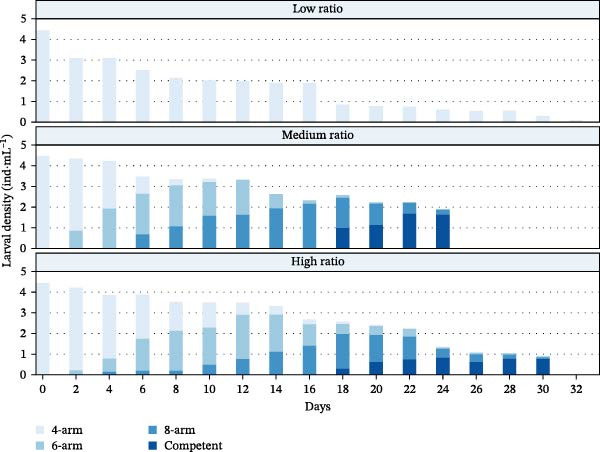
Temporal variation in larval concentration (individuals·mL^−1^) of *Paracentrotus lividus* at different developmental stages under low (LR), medium (MR), and high (HR) food ratio conditions. Stacked bars represent mean values calculated across replicate cultures at each sampling day. Bluescale shading indicates developmental stage (4‐arm, 6‐arm, 8‐arm pluteus, and competent larvae). Two‐way PERMANOVA results assessing the effects of time (df = 16, *F* = 105.9, *p*  < 0.001), food ratio (df = 2, *F* = 159.4, *p*  < 0.001), and their interaction (df = 32, *F* = 12.7, *p*  < 0.001) on the number of *P. lividus* larvae at different development stages across three food ratio conditions (low, medium, and high). Detailed bar plots including standard deviations are provided in Figure S2.

Larval development, survival, and age‐at‐competence were all affected by the microalgal diets (low, medium, and high ratio, Table 1). As shown in Figure 1, the larvae fed with medium ratio obtained the fastest development, arriving at the 8‐arm stage in only 14 days, followed by the larvae fed with high ratio after 16 days. Instead, larvae fed with low ratio never reached the 6‐arm pluteus stage and never developed into settled juveniles, remaining in the 4‐arm stage until the end of the experiment. The rudiment was observed for the first time after 14 days for larvae fed with medium ratio, and the competence (50% of pluteus with well‐developed rudiment) was reached after 20 days. Instead, in larvae fed with the high ratio, the first appearance of the rudiment and the competence were delayed compared to the medium ratio, respectively, after 18 and 24 days. Moreover, the larval development in high ratio was more irregular. In this condition, 4‐arm larvae and 6‐arm larvae were detected up to 26 and 30 days, leading to a more asynchronous turnover among the development stages (Figure 1). In fact, although the first appearance of 8‐arm larvae occurred in the first days of rearing (after 4 days), the transition of larval cultures to this stage (at least 50% of larvae) was not reached until the 18th day.

**Figure 2:**
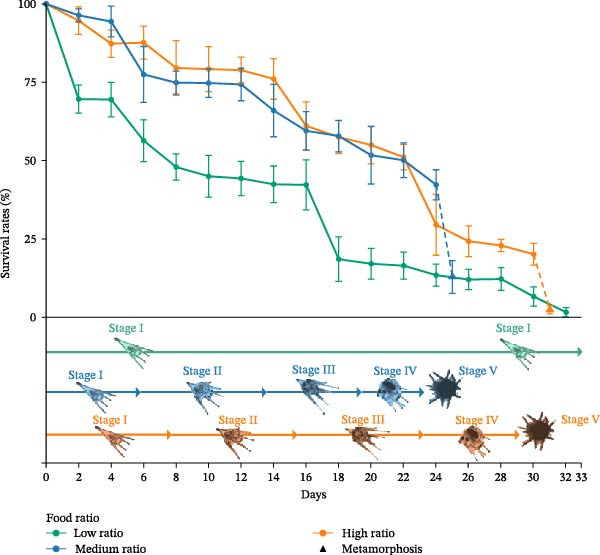
Survival rate of the *Paracentrotus lividus* larvae fed with the three food ratio conditions (low, medium and high ratios) over the experimental time until achieving the competence stage. Final metamorphosis yields (%) at the end of the experiment are shown for each treatment. Values represent mean ± SD of three replicate cultures. The lower panel illustrates the corresponding developmental progression under each feeding regime. Two‐way PERMANOVA results assessing the effects of time (df = 16, *F* = 208.7, *p*  < 0.001), food ratio (df = 2, *F* = 395.6, *p*  < 0.001), and their interaction (df = 32, *F* = 1.94, *p*  > 0.05) on larval survival rate at different development stages across three food ratio conditions (low, medium, and high).

Larval density progressively decreased throughout the experiment as a result of mortality. Consequently, temporal changes in larval concentration among treatments reflected treatment‐specific survival patterns. The survival rates of larvae and metamorphosis yields (ratio of larvae that metamorphosed into juveniles) in the three microalgae concentration conditions are shown in Figures 1 and 2. Larvae fed with the medium ratio achieved the highest final survivorship (42.4 %) and metamorphosis rate (13.1% of the initial larvae), although no significant differences emerged in the survival rate between medium and high ratio conditions for the first 22 days. After this period, high mortality rates were observed in tanks fed with the high ratio, where a final survival rate of 20.3% and a metamorphosis rate of 2.6% were detected at the end of the experiment. Independent samples *t*‐tests confirmed significant differences between the medium ratio and high ratio treatments in both final survivorship and metamorphosis rates, with large effect sizes according to Cohen’s *d* (Table 2). In the low ratio condition instead, all larvae sank at the end of the experiment without reaching the competence stage (or even the 6‐arm pluteus stage).

**Table 2:** Independent samples *t*‐tests comparing final survivorship and metamorphosis rates of sea urchin larvae fed high‐ratio (HR) and medium‐ratio (MR) algal diets.

Larval performance metric	HR		MR						
	Mean	SD	Mean	SD	df	*t*‐test	*p*‐value	Cohen’s *d*	*F*‐value
Survivorship	20.38	4.61	42.17	6.02	2	4.98	0.007	2.55	1.70
Metamorphosis	2.57	1.18	13.06	4.46	2	2.77	0.018	3.16	14.19

*Note:* Cohen’s *d* indicates the magnitude of differences between groups. *F*‐statistics compare variances between groups. Significance levels:  ^∗^ *p* < 0.05,  ^∗^ *p* < 0.01.

## 4. Discussion

Food quality is generally considered the main factor affecting the development and the metamorphosis in planktotrophic larval rearing [26, 33, 48]. However, this study also highlighted the pivotal role of food quantity in determining larval yields.

We utilized a mix of three microalgae strains, known for their optimal results in previous investigations with *P. lividus* [10, 28, 29, 32]. These strains differ markedly in cell size and biochemical composition, thereby providing a nutritionally balanced diet throughout larval development. The cryptophyte *R. lens* (≈20 ± 2 µm in diameter) is characterized by a high content of essential fatty acids and nutrients, with lipids accounting for ~10%–30% and proteins for 30%–60% of dry weight [34]. The diatom *C. calcitrans* (7.3 ± 0.8 µm) is known for its high protein and lipid content and for elevated levels of polyunsaturated fatty acids, particularly a high EPA/ARA ratio [49]. The haptophyte *I. galbana* (≈5 ± 1 µm in diameter) further contributes a substantial lipid fraction (20%–30% w/dw) and represents an important source of ω‐3 PUFAs [49, 50]. The microalgal size range (from 5 to 20 µm) and nutritional profiles well meet the *P. lividus* dietary requirements during the larval stage [34, 49, 50], being easily ingestible from echinopluteus, which presents an oral structure that ranges between 20–100 µm depending on the developmental stage [35].

However, in addition to confirming the crucial role of a suitable mixed‐algae diet, the results of this investigation clearly highlighted the importance of food quantity for *P. lividus* larval development and the existence of a specific threshold that these planktotrophic larvae can efficiently assimilate. In order to isolate the effect of food quantity, algal species, culture conditions, and harvesting procedures were kept constant across treatments, ensuring comparable nutrient compositions among rations. Therefore, differences observed among treatments can be attributed primarily to changes in total nutrient availability driven by the feeding rate, rather than to variations in diet quality. Moreover, as larval density declined during the trial, effective food exposure per larva increased progressively over time despite constant administered concentrations at each developmental stage. This dynamic shift in per‐capita food availability (Table S1) should therefore be considered when interpreting treatment differences, particularly at later developmental stages.

Among the three food ratios analyzed, the medium ratio (1500 cells·mL^−1^ for 4‐arm larvae, 3000 cells·mL^−1^ for 6‐arm larvae, and 6000 cells·mL^−1^ for 8‐arm larvae) showed the highest performances in terms of both survival rates and time to reach the competence. This pattern was also corroborated by the difference in metamorphosis yield obtained from the experimental diets. The percentage of metamorphized larvae in the medium ratio condition was the highest, highlighting that this food quantity provided adequate energy to complete the larval development and metamorphosis in benthic juveniles. Similar results were previously reported for other sea urchin species. For example, Kelly et al. [51] detected that larvae of *Psammechinus miliaris* fed with a 1500–4000 cells·mL^−1^ ratio of *Hymenomonas elongata* showed a higher metamorphosis success, conversely to larvae fed with a lower ratio of 500 cells·mL^−1^. In another study on *Echinus esculentus*, Jimmy et al. [52] observed that a ratio between 1000 and 5000 cells·mL^−1^ of *Dunaliella tertiolecta* produced better results in larval rearing than a ratio of 3000–15,000 cells·mL^−1^. Azad et al. [25] observed that larvae of *S. purpuratus* fed with a ratio from 2.5 × 10^3^ to 10.0 × 10^3^ cells·mL^−1^ was the only condition to reach the competence and metamorphosis, whereas larvae fed with lower or higher concentrations failed to attain competence by the end of the experiment. Together, these studies indicate that planktotrophic echinoid larvae may perform optimally within a restricted food‐ration window, with both food limitation and food excess impairing development. In this context, the present study extends this concept to *P. lividus* by providing, to our knowledge, the first systematic and quantitative assessment of stage‐specific feeding ranges under controlled hatchery conditions. Our results indicate the existence of an optimal food ratio for this species, with both suboptimal and excessive rations compromising larval development, survival, and metamorphosis success. This finding suggested that the different food quantities could play a key role in larval ontogenetic development by reflecting the species’ life‐history traits. In line with this hypothesis, Fenaux et al. [53] and Present et al. [54] claimed that the effects of food‐limited and food‐surplus conditions in planktonic larvae of benthic invertebrates may have important implications for their ecology and evolution. Cocurullo et al. [36] reported that *P. lividus*, *A. lixula*, and *S. purpuratus* larvae adjust arm morphology in response to food availability, with fed larvae developing shorter postoral rods than starved larvae under light–dark conditions. This supports the interpretation that food availability is a developmental cue capable of altering larval morphology, energy allocation, growth, and survivorship in echinoids. Although direct morphometric indices, such as arm length and body width, were not measured in the present study, the combined assessment of developmental progression, survival, competence achievement, and metamorphosis success provided an integrative evaluation of larval performance across the whole rearing cycle. Therefore, potential sublethal effects on larval condition were considered in terms of their functional consequences for development and successful transition to the juvenile stage. Indeed, pluteus fed with low ratio remained in the 4‐arm stage for the whole duration of the experiment (33 days), proving that limiting supplies of food lead to the planktonic period extension. A comparable developmental bottleneck was reported for the New Zealand sea urchin *Evechinus chloroticus*, in which field larvae accumulated at the late 4‐arm pluteus stage, spent 44.9% of their development time in this stage, and only 11.5% survived beyond it, a pattern interpreted as evidence of food limitation in the plankton [55]. Similar responses have also been described in other planktotrophic marine larvae, although the specific developmental bottleneck may differ among taxa. For example, low food availability delays growth and metamorphosis in bivalve larvae, with *Macoma balthica* metamorphosing later and at a smaller size under low‐food conditions [56], while food limitation in crustacean larvae can prolong larval development and reduce survival or body condition at metamorphosis [57]. Therefore, the prolonged persistence of larvae at the 4‐arm stage under the low ratio treatment may be interpreted as a form of developmental plasticity in response to food limitation. In planktotrophic marine invertebrates, delaying developmental progression under poor trophic conditions may have potential adaptive significance because it can extend the pelagic period and increase the probability that larvae encounter more favorable feeding environments before reaching competence. This interpretation is consistent with recent integrative evidence from sea urchin larvae showing that food deprivation does not necessarily result in immediate developmental failure but may activate resilience mechanisms that allow larvae to maintain physiological functions and recover when algal food becomes available. For example, Li et al. [58] showed that unfed *S. purpuratus* larvae survived prolonged algal food deprivation, maintained respiration and protein turnover, conserved biochemical reserves, and were able to recover after delayed feeding. Such findings support the view that food‐limited echinoid larvae can display considerable physiological and developmental resilience.

However, this plastic response may also entail important costs, including prolonged exposure to planktonic mortality, delayed settlement, reduced energetic condition, and lower probability of successful metamorphosis if food limitation persists. In the present study, larvae exposed to the low ratio remained at the 4‐arm stage throughout the experimental period and failed to reach competence, suggesting that the observed delay reflected an energetic bottleneck under the tested rearing conditions rather than successful compensatory development. Future studies should determine whether increases in food availability at specific developmental stages can rescue development or whether irreversible thresholds exist beyond which recovery is no longer possible. In the high ratio condition, on the other hand, the larvae developed quickly in the first days of the experiment, with the first appearance of 8‐arm pluteus after just 4 days. However, in this condition, the ages of competence and metamorphosis were reached later than in the medium ratio, and the survival rate was also significantly lower. Fenaux et al. [53] suggested that *P. lividus* larvae have an upper limit on the rate of ingestion of food particles. In this study, we expanded this concept, showing that the higher food ratio could have a negative impact on the larval development. In the high ratio treatment, food administration reached up to 3.59 µg·larva^−1^ at later stages, compared to 0.51 µg·larva^−1^ in the medium ratio treatment, indicating a markedly higher per‐capita nutritional load (Table S1). Considering the conspicuous daily water exchange in the culture, this result could be related to the accumulation in larval bodies of harmful catabolites due to the acceleration of their metabolism. Thorson [59] claimed that the amount of food consumed by marine invertebrate larvae is five times that of an adult per unit of weight and time. This adaptation strategy allows the optimization of food intake in low‐nutrient environments but may also increase larval metabolic efforts, potentially leading to the production of harmful compounds, such as reactive oxygen species (ROS), which could ultimately impact their survival [60]. Therefore, further investigations based on the metabolomic and transcriptomic approaches will be necessary to fully clarify the mechanisms at the basis of larval ingestion, digestion, and ontogenesis.

This study also pointed out the effectiveness of two methodological advances in the production of sea urchin juveniles. First, considering the importance of food amount in larval development, our results highlighted the necessity of using a reliable microalgae quantification method in *P. lividus* hatchery production to provide the appropriate food ratio, which must vary depending on the larval stage. The spectrophotometry methodology adopted in the present study allowed to define the relationship among OD, cell density, and dry biomass of algal culture, which in turn provided a simple, time‐saving, and standardizable method for quantifying the volume of each algae to be daily administrated to the pluteus, conversely to the traditional cell counting, which is a laborious, error‐prone, and inaccurate method [61]. Second, this study evidenced the advantages of a noninvasive gathering method of the metamorphosized individuals. KCl solution 0.5% (weight/volume) emerged as an efficient and nontoxic agent for inducing paralysis and detachment in *P. lividus* juveniles, and it can replace the time‐consuming and inefficient manual collecting techniques. In this way, mechanical damage caused to the delicate podia and epithelium of juvenile sea urchins by the use of a brush, forceps, or water jet was prevented. The introduction of this noninvasive detachment technique may significantly improve the performance in hatcheries. In fact, handling of juveniles in aquaculture applications is a pivotal activity. However, the manual detachment method is an inevitably damaging operation that leads to tube feet and other tissue broken in juveniles. Additionally, postmetamorphosis individuals exhibit cryptic behavior that makes them difficult to collect manually. In this regard, further studies are needed to evaluate the survival rate of *P. lividus* juveniles detached using the KCl method in the days following paralysis induction.

To summarize, the present study evidenced that the food ratio has a pivotal role in *P. lividus* larval rearing performances and must be considered to achieve the economic effectiveness of large‐scale hatchery production. Practical guidelines for optimizing sea urchin larval rearing can be established by integrating our results in terms of survival rate, timing of first transition to next development stage, and timing of transition to next stage in 50% of larvae (Table 3). During the first 16 days, larvae fed with MR exhibited the fastest development, reaching the transition to the 8‐arm stage. However, at the same time, larvae fed with HR achieved higher survival rates, with some individuals reaching the 8‐arm stage as early as 4 days. After this period, the MR resulted always the best performing treatment for the 8‐arm and competence stages.

**Table 3:** Best performing ratio (µg·mL^−1^ and cells·mL^−1^) and recommended food ratio ranges (µg·mL^−1^ and cells·mL^−1^) for each *P. lividus* larval stage, defined based on total survival, first transition to next stage (FT), transition to next stage in 50% of larvae (T50).

Developmental stage	Best performing ratio	Recommended range
4‐arms	0.40 µg·mL^−1^ 5000 cells·mL^−1^	0.12–0.40 µg·mL^−1^ 1500–5000 cells·mL^−1^
6‐arms	0.80 µg·mL^−1^ 10,000 cells·mL^−1^	0.24–0.80 µg·mL^−1^ 3000–10,000 cells·mL^−1^
8‐arms	0.48 µg·mL^−1^ 6000 cells·mL^−1^	0.48 µg·mL^−1^ 6000 cells·mL^−1^
Competence	0.96 µg·mL^−1^ 12,000 cells·mL^−1^	0.96 µg·mL^−1^ 12,000 cells·mL^−1^

*Note:* Recommended ranges were defined under the experimental rearing conditions used in the present study, with larval densities of ~4–3 larvae·mL^−1^ in early (4‑arm and 6‑arm) stages and 2–2.5 larvae·mL^−1^ in later (8‑arm and competent) stages. For detailed table see Table S2.

In conclusion, the ontogenetic development of *P. lividus* is highly dependent on food availability. Therefore, scientific research on the optimal feeding regime for planktonic larvae is crucial for understanding the implications of both food‐limited and food‐surplus conditions on life‐history traits, species ecology, and aquaculture requirements.

In nature, the dynamics of phytoplankton are fundamental in determining the health and biodiversity of marine ecosystems. Any disruption at this food web level can have cascading effects on higher trophic levels, including zooplankton and marine megafauna [62]. In the Mediterranean region, the IPCC report (Intergovernmental Panel on Climate Change, 2023) projects a 4% decrease in precipitation per 1°C of global warming, with a huge impact on marine ecosystem productivity, consistent with modeling that links reduced river flow and climatic forcing to lower primary production and consumer biomass in the basin [63–65]. Such changes are expected to directly affect phytoplankton abundance, composition, and temporal availability, potentially increasing the frequency of food‐limited conditions for planktotrophic larvae. Given these potential changes, it is essential to investigate how food availability may influence the development of marine invertebrates, particularly during larval stages in dynamic environments [58, 66–68]. In this context, our findings, although primarily focused on artificial conditions, contribute to the understanding of benthic invertebrate meroplanktonic patterns in natural environments by experimentally defining food quantity thresholds beyond which larval development may be delayed or impaired. While extensive research exists on *P. lividus* aquaculture, this study further advances the field of sea urchin hatchery practices. By fine‐tuning feeding strategies, we can improve growth rates and survival in captivity, ultimately reducing pressure on wild sea urchin populations harvested for consumption. This sustainable approach supports conservation efforts while enhancing the long‐term viability of industries dependent on these species.

## Author Contributions

**Luca Grosso**: conceptualization, methodology, investigation, statistical analysis, writing – first draft of the manuscript. **João Sousa, Pedro M. Santos**, **and Francisco Azevedo e Silva**: investigation, manuscript revision. **Sílvia Lourenço, Pedro M. Félix, Ana Pombo**: writing – sections of the manuscript, manuscript revision, reading, approval of the submitted version, conceptualization, supervision, project administration. **Arnold Rakaj**: conceptualization, methodology, investigation, resources, writing, project administration, validation, supervision.

## Funding

This work was supported by the EMBO Scientific Exchange Grant (10285), the INTEGMED project (PO FEAMP, Measure 2.47; Grant J89J19000920005; 2019), and the Portuguese Foundation for Science and Technology (FCT). FCT support was provided through the strategic Projects UIDB/04292/2020 and UIDP/04292/2020, granted to MARE—Marine and Environmental Sciences Centre, and through Project LA/P/0069/2020, granted to the Associate Laboratory ARNET—Aquatic Research Network.

## Conflicts of Interest

The authors declare no conflicts of interest.

## Supporting Information

Additional supporting information can be found online in the Supporting Information section.

## Supporting information


**Supporting Information** Figure S1: The representative developmental stages of *Paracentrotus lividus*, from fertilized egg to juvenile stage, and supports the identification of the larval and postmetamorphic stages described in Section 2. Figure S2: Detailed bar plots, including standard deviations, of larval concentration at different developmental stages under the three feeding regimes. This figure complements Figure 1 by showing the variability among replicate cultures throughout the experimental period. Table S1: Larval density, administered food concentration, and the corresponding effective food availability per larva for each sampling day under the three experimental feeding treatments. This table provides additional information supporting the description of the initial larval densities and the interpretation of per‐capita food availability during the experiment. Table S2: The detailed recommended food‐ratio ranges for each *P. lividus* larval stage, defined according to total survival, first transition to the next developmental stage, and transition to the next stage in 50% of larvae. This table provides additional details supporting the recommended feeding ranges summarized in Table 3.

## Data Availability

The data that support the findings of this study are available from the corresponding author upon reasonable request.
