# Supplementary material for: Excess Food Availability Can Have Detrimental Effects on Planktotrophic Larvae: Evaluation of the Quantitative Feeding on Sea Urchin Paracentrotus lividus Larval Rearing
Source: Aquac Nutr. 2026 Jul 27;2026:6467396. doi: 10.1155/anu/6467396 (PMC13403033; doi:10.1155/anu/6467396)
Supplement: Supplementary file 1 — Supporting Information Figure S1: The representative developmental stages of Paracentrotus lividus, from fertilized egg to juvenile stage, and supports the identification of the larval and postmetamorphic stages described in Section 2. Figure S2: Detailed bar plots, including standard deviations, of larval concentration at different developmental stages under the three feeding regimes. This figure complements Figure 1 by showing the variability among replicate cultures throughout the experimental period. Table S1: Larval density, administered food concentration, and the corresponding effective food availability per larva for each sampling day under the three experimental feeding treatments. This table provides additional information supporting the description of the initial larval densities and the interpretation of per‐capita food availability during the experiment. Table S2: The detailed recommended food‐ratio ranges for each P. lividus larval stage, defined according to total survival, first transition to the next developmental stage, and transition to the next stage in 50% of larvae. This table provides additional details supporting the recommended feeding ranges summarized in Table 3. [file ANU-2026-6467396-s001.docx]

**Supplementary Materials**

**Supplementary Figure S1.** Representative developmental stages of Paracentrotus lividus: fertilized egg (a), 2-cell stage (b), 4-cell stage (c), morula/blastula stage (d–e), gastrula/prism stage (f), 4-arm pluteus (g), 6-arm pluteus (h), 8-arm pluteus (i), competent larva (j), early post-metamorphic juvenile (k), and juvenile stage (l). Scale bars are shown in each panel. Images are intended to support visual developmental stage identification and were not used for morphometric measurements.

**
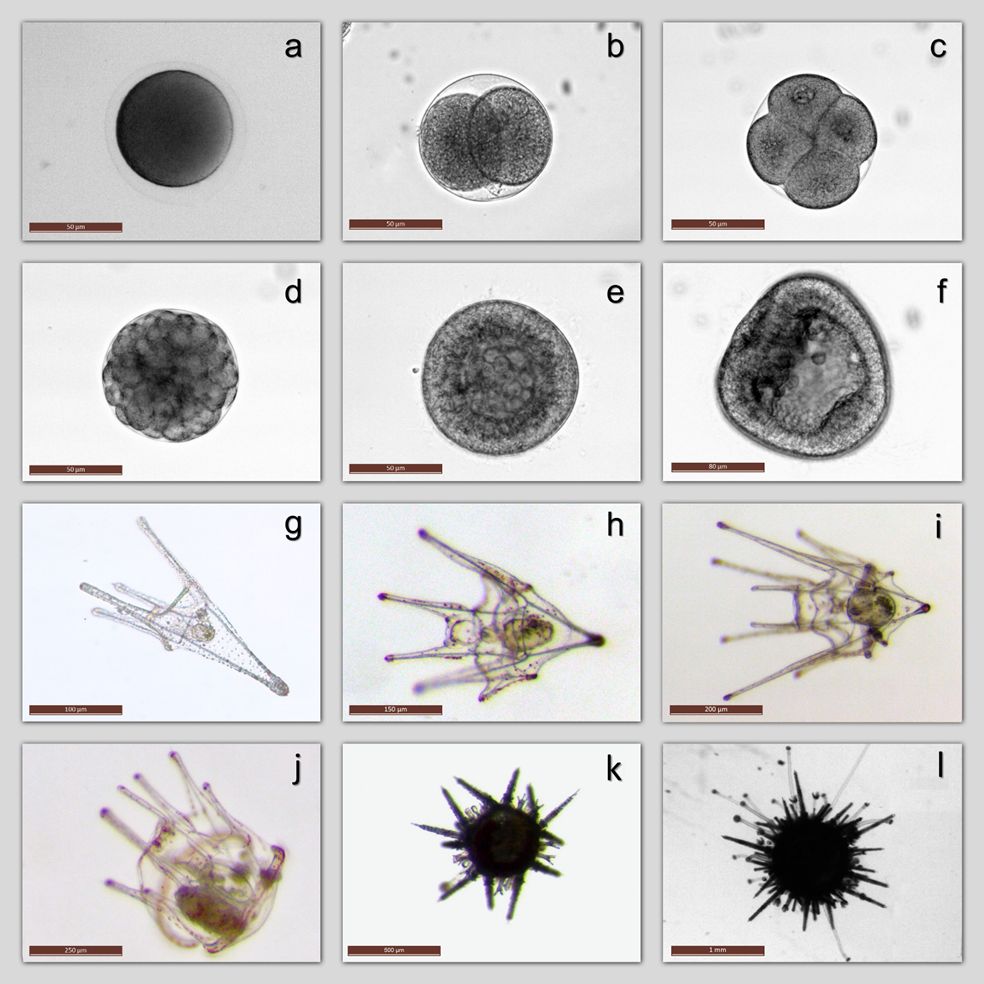
**

**Supplementary Figure S2.** Detailed bar plots including standard deviations of larval concentration (individuals·mL⁻¹) of Paracentrotus lividus at different developmental stages under Low (LR), Medium (MR), and High (HR) food ratio conditions throughout the experimental time. Bars represent mean values calculated across replicate cultures at each sampling day and the grayscale shading indicates developmental stage (4-arm, 6-arm, 8-arm pluteus and competent larvae). Two-way PERMANOVA results assessing the effects of time (df = 16, F = 105.9, p < 0.001), food ratio (df = 2, F = 159.4, p < 0.001), and their interaction (df = 32, F = 12.7, p < 0.001) on the number of P. lividus larvae at different development stages across three food ratio conditions (Low, Medium, High).


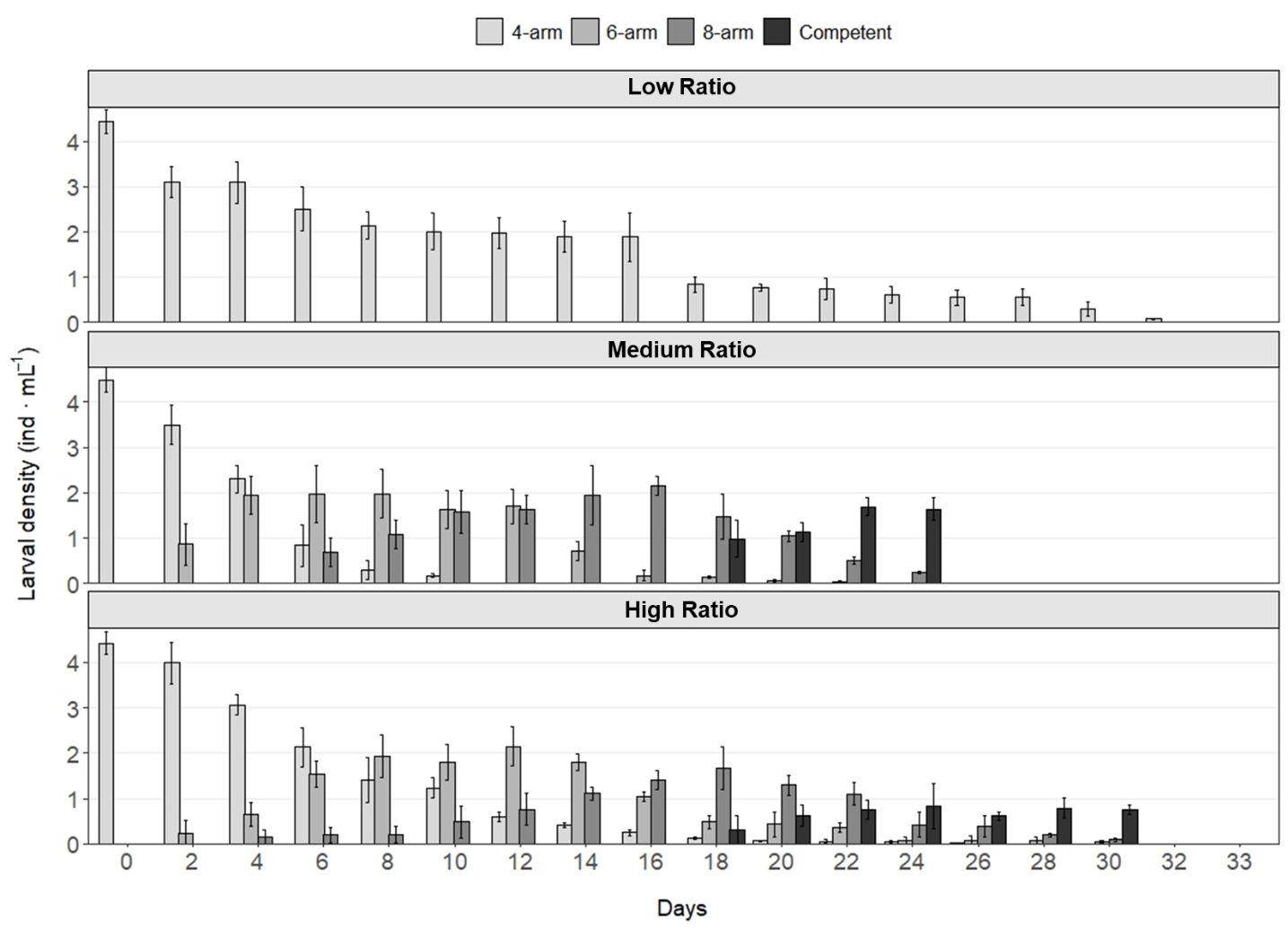


**Supplementary Table S1.** Larval density (ind·mL⁻¹), administered food concentration (µg DW·mL⁻¹ and cells·mL⁻¹), and corresponding effective food availability per larva (µg DW·larva⁻¹ and cells·larva⁻¹) for each sampling day under the three feeding treatments: Low Ratio, Medium Ratio, and High Ratio. Effective food availability was calculated as administered food concentration divided by observed larval density at each time point

| Days | **Treatment** | **Larval density (ind·mL⁻¹)** | **Food concentration (µgDW·mL⁻¹)** | **Food concentration (cells·mL⁻¹)** | **Effective food availability (µgDW·larva⁻¹)** | **Effective food availability (cells·larva⁻¹)** |
| --- | --- | --- | --- | --- | --- | --- |
| 0 | LR | 4.44 | 0.04 | 500 | 0.01 | 112.69 |
| 2 | LR | 3.10 | 0.04 | 500 | 0.01 | 161.47 |
| 4 | LR | 3.09 | 0.04 | 500 | 0.01 | 161.70 |
| 6 | LR | 2.51 | 0.04 | 500 | 0.02 | 199.06 |
| 8 | LR | 2.14 | 0.04 | 500 | 0.02 | 233.68 |
| 10 | LR | 2.01 | 0.04 | 500 | 0.02 | 248.41 |
| 12 | LR | 1.98 | 0.04 | 500 | 0.02 | 252.49 |
| 14 | LR | 1.89 | 0.04 | 500 | 0.02 | 263.92 |
| 16 | LR | 1.89 | 0.04 | 500 | 0.02 | 264.46 |
| 18 | LR | 0.84 | 0.04 | 500 | 0.05 | 593.34 |
| 20 | LR | 0.77 | 0.04 | 500 | 0.05 | 645.72 |
| 22 | LR | 0.74 | 0.04 | 500 | 0.05 | 672.16 |
| 24 | LR | 0.61 | 0.04 | 500 | 0.07 | 820.89 |
| 26 | LR | 0.55 | 0.04 | 500 | 0.07 | 912.51 |
| 28 | LR | 0.55 | 0.04 | 500 | 0.07 | 902.46 |
| 30 | LR | 0.31 | 0.04 | 500 | 0.13 | 1630.81 |
| 32 | LR | 0.08 | 0.04 | 500 | 0.49 | 6068.17 |
| 0 | MR | 4.48 | 0.12 | 1500 | 0.03 | 334.56 |
| 2 | MR | 4.35 | 0.12 | 1500 | 0.03 | 345.09 |
| 4 | MR | 4.23 | 0.12 | 1500 | 0.03 | 354.89 |
| 6 | MR | 3.48 | 0.24 | 3000 | 0.07 | 862.84 |
| 8 | MR | 3.35 | 0.24 | 3000 | 0.07 | 896.24 |
| 10 | MR | 3.37 | 0.24 | 3000 | 0.07 | 889.78 |
| 12 | MR | 3.32 | 0.24 | 3000 | 0.07 | 904.59 |
| 14 | MR | 2.63 | 0.48 | 6000 | 0.18 | 2279.01 |
| 16 | MR | 2.32 | 0.48 | 6000 | 0.21 | 2582.93 |
| 18 | MR | 2.58 | 0.48 | 6000 | 0.19 | 2324.87 |
| 20 | MR | 2.23 | 0.96 | 12000 | 0.43 | 5382.93 |
| 22 | MR | 2.23 | 0.96 | 12000 | 0.43 | 5375.52 |
| 24 | MR | 1.88 | 0.96 | 12000 | 0.51 | 6396.35 |
| 0 | HR | 4.43 | 0.4 | 5000 | 0.09 | 1128.38 |
| 2 | HR | 4.21 | 0.4 | 5000 | 0.09 | 1186.54 |
| 4 | HR | 3.85 | 0.4 | 5000 | 0.10 | 1299.09 |
| 6 | HR | 3.88 | 0.4 | 5000 | 0.10 | 1290.08 |
| 8 | HR | 3.53 | 0.8 | 10000 | 0.23 | 2833.56 |
| 10 | HR | 3.51 | 0.8 | 10000 | 0.23 | 2850.69 |
| 12 | HR | 3.49 | 0.8 | 10000 | 0.23 | 2866.99 |
| 14 | HR | 3.32 | 0.8 | 10000 | 0.24 | 3016.19 |
| 16 | HR | 2.68 | 1.6 | 20000 | 0.60 | 7461.33 |
| 18 | HR | 2.58 | 1.6 | 20000 | 0.62 | 7762.37 |
| 20 | HR | 2.42 | 1.6 | 20000 | 0.66 | 8280.60 |
| 22 | HR | 2.27 | 1.6 | 20000 | 0.71 | 8828.06 |
| 24 | HR | 1.36 | 3.2 | 40000 | 2.34 | 29309.08 |
| 26 | HR | 1.08 | 3.2 | 40000 | 2.95 | 36916.40 |
| 28 | HR | 1.04 | 3.2 | 40000 | 3.08 | 38481.24 |
| 30 | HR | 0.89 | 3.2 | 40000 | 3.59 | 44847.67 |

**Supplementary Table S2.** Recommended food ratio ranges for each P. lividus larval stage, defined based on total survival, first transition to next stage (FT), transition to next stage in 50% of larvae (T50). Colours represent indicator performances: Best performing condition in green; intermediate condition in yellow; worst performing condition in red.

| **Developmental stage** | **Comparison indicators** | **Low ratio diet (cell/mL provided)** | **Medium ratio diet (cell/mL provided)** | **High ratio diet (cell/mL provided)** | **Best performing ratio (cell/mL)** | **Recommended range (cell/mL)** |
| --- | --- | --- | --- | --- | --- | --- |
| 4-arms | Total Survival | 500 | 1500 | 5000 | 5000 | 1500-5000 |
|  | First Transition to next stage | 500 | 1500 | 5000 |  |  |
|  | Transition to next stage in 50% of larvae | 500 | 1500 | 5000 |  |  |
| 6-arms | Total Survival | 1500 | 3000 | 10000 | 10000 | 3000-10000 |
|  | First Transition to next stage | 1500 | 3000 | 10000 |  |  |
|  | Transition to next stage in 50% of larvae | 1500 | 3000 | 10000 |  |  |
| 8-arms | Total Survival | 3000 | 6000 | 20000 | 6000 | 6000 |
|  | First Transition to next stage | 3000 | 6000 | 20000 |  |  |
|  | Transition to next stage in 50% of larvae | 3000 | 6000 | 20000 |  |  |
| Competence | Total Survival | 6000 | 12000 | 40000 | 12000 | 12000 |
|  | First Transition to next stage | 6000 | 12000 | 40000 |  |  |
|  | Transition to next stage in 50% of larvae | 6000 | 12000 | 40000 |  |  |
